# Alleviation of thalamic pain by cilostazol administration: a case report

**DOI:** 10.1002/ccr3.1363

**Published:** 2018-01-10

**Authors:** Ayaka Haruta‐Tsukamoto, Hideki Funahashi, Yu Miyahara, Tomoko Matsuo, Toshikazu Nishimori, Yasushi Ishida

**Affiliations:** ^1^ Department of Psychiatry Faculty of Medicine University of Miyazaki 5200 Kihara, Kiyotake Miyazaki‐city Miyazaki 889‐1692 Japan

**Keywords:** Cilostazol, exchange protein directly activated by cAMP, phosphodiesterase 3A, thalamic pain

## Abstract

Thalamic pain is severe and treatment‐resistant; however, there are few available options for improving thalamic pain. This study demonstrated that thalamic pain was alleviated by administration of cilostazol, suggesting that cilostazol may be a candidate for treating thalamic pain.

## Introduction

Thalamic pain was first described by Dejerine and Roussy 1 as, “…severe, persistent, paroxysmal, often intolerable, pains on the hemiplegic side, not yielding to any analgesic treatment” 2. Indeed, thalamic pain is characterized by the appearance of constant or intermittent pain in the hemibody, contralateral to the thalamic lesion, and is a severe and treatment‐resistant type of central post‐stroke pain (CPSP) that occurs after a cerebrovascular lesion results in thalamic stroke. Therefore, most of relatively few studies of treatment that have been published cannot claim the gold standard of the randomized, controlled trial 3. Treating CPSP remains a challenge; however, the pharmacological options include antidepressants, antiepileptics, opioids, NMDA‐receptor antagonists, and antiarrhythmics. For example, amitriptyline, a tricyclic antidepressant, is usually used as a drug of first choice for CPSP 4, 5. Nevertheless, the recent evaluated therapies, including amitriptyline, have been largely unsuccessful in controlling CPSP 6, 7, indicating that few options are available for improving CPSP.

Central post‐stroke pain is considered to be persistent neuropathic pain of central origin that is generated after stroke. Some anticoagulants and antiplatelets, including triflusal, cilostazol, sarpogrelate, and miscellaneous drugs, may be effective for secondary stroke prevention 8. Cilostazol is also a drug of first choice for intermittent claudication in addition to secondary prevention of cerebral infarction 9, suggesting that the treatment of cilostazol may result in improvement in cerebral infarction. However, there have been no reports on the effects of cilostazol in patients with thalamic pain derived from cerebral infarction. Thus, cilostazol was administered to a patient with both thalamic pain and intermittent claudication, and its effect on thalamic pain was mainly evaluated.

## Case Description

The patient was a 66‐year‐old man with a history of hypertension, hypertensive retinopathy, severe pneumonia, and lacunar infarction. Three years previously, a left‐side thalamic hemorrhage developed suddenly, and he experienced persistent pain and numbness of the right upper and lower limbs. Just before treatment, pain and numbness of the right side of face, upper and lower limbs, and intermittent claudication emerged. At the start of treatment, the visual analogue scale (VAS) without walking was 92, indicating severe pain, while he complained about pain in both legs after walking. Additionally, the dorsal arteries of both feet were impalpable, and he had depression, thalamic syndrome, hypertension, and probable arteriosclerosis obliterans. His score on the 21‐item Hamilton Rating Scale for Depression (HAMD‐21) was 20, indicating severe depression.

**Figure 1:**
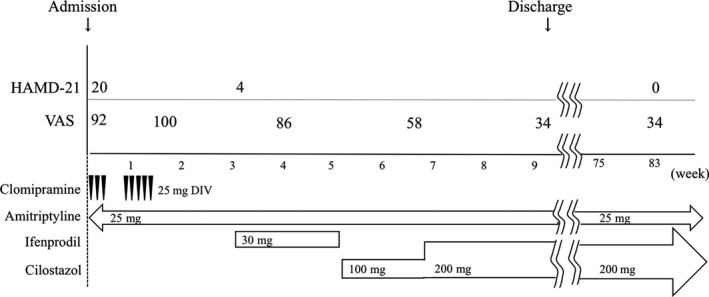
The clinical and therapeutic course of the patient. HAMD‐21, 21‐item Hamilton Rating Scale for Depression; VAS, visual analogue scale; DIV, intravenous drip infusion. The pain score (VAS) was decreased after the start of cilostazol treatment.

Amitriptyline (25 mg/day), a tricyclic antidepressant, was administered throughout treatment and is currently still administered. Clomipramine (25 mg/day), another tricyclic antidepressant, was administered in addition to amitriptyline by intravenous drip for 8 days. Consequently, his HAMD‐21 score was reduced to 4. No noticeable changes in the VAS or intermittent claudication were observed. Thereafter, ifenprodil (30 mg/day), a cerebral circulation activator, was administered in addition to amitriptyline for 18 days. The VAS and intermittent claudication showed little change. Thus, ifenprodil was switched to cilostazol that has an antiplatelet effect on the cerebral circulation, and is thus widely used to treat intermittent claudication. Treatment with cilostazol (100 mg/day) for 1 week gradually improved the intermittent claudication and VAS score; indeed, the VAS score improved by 58 points, indicating mid‐level pain. The administration of cilostazol (200 mg/day) for 2 weeks moreover improved the intermittent claudication, and he could walk and stretch voluntarily. Concurrently, his VAS score improved furthermore by 30 points, indicating slight pain (Fig. 1), and he was discharged from the hospital. He is currently continuing treatment as an outpatient and is receiving cilostazol and amitriptyline (Fig. 1).

**Figure 2:**
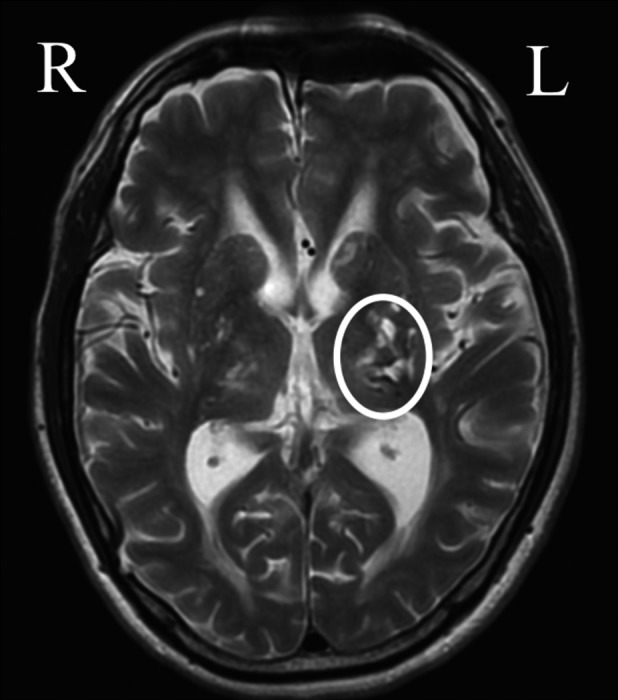
T2‐weighted MRI imaging of brain. Obsolete cerebral infarction was recognized in the left‐dominated thalamus as illustrated by a circle.

**Figure 3:**
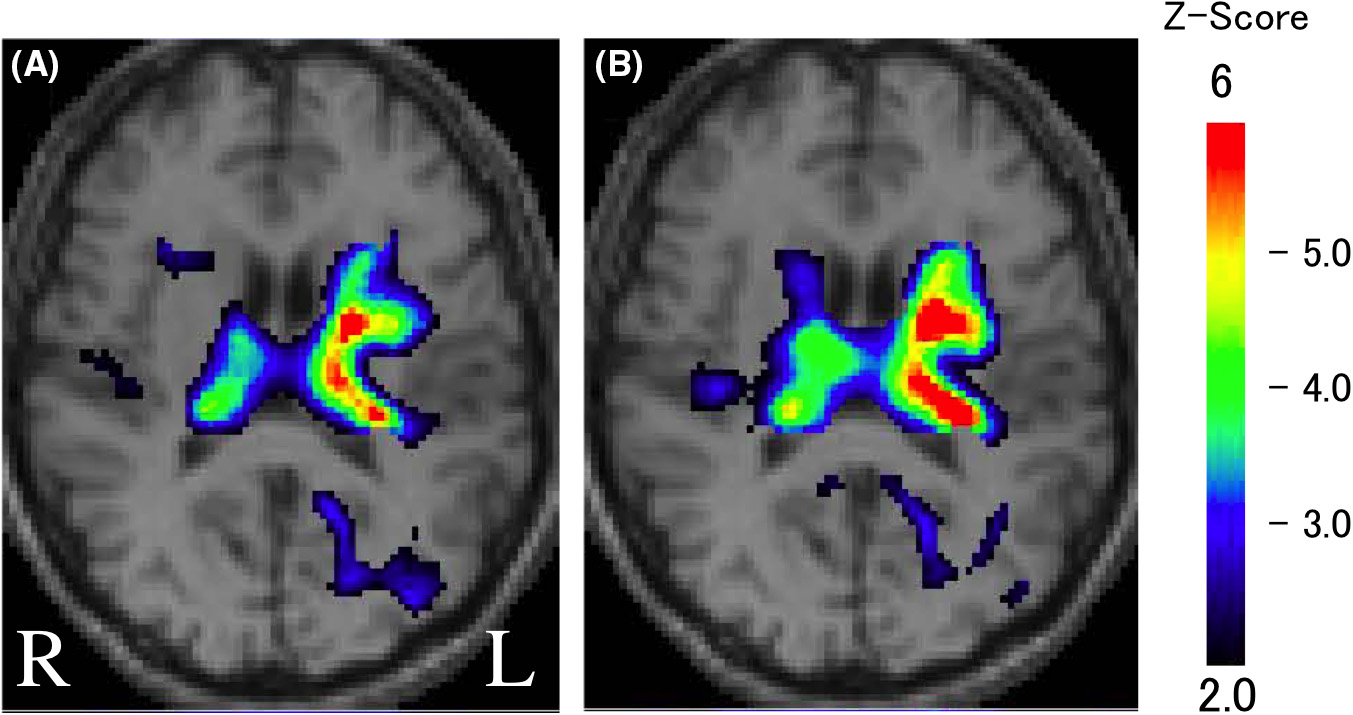
^99m^Tc‐ECD SPECT with the easy Z‐score imaging system findings from August 2015 (A) and January 2017 (B). A left‐dominated reduction of cerebral blood flow was recognized in the thalamus before (A) and after treatment with cilostazol (B).

T2‐weighted magnetic resonance imaging (MRI) of the brain revealed a clear lesion derived from an obsolete hemorrhage, predominantly in the left thalamus (Fig. 2). Concurrently, ^99m^Tc‐ethylene cysteine diethylester (ECD) single‐photon emission computed tomography (SPECT) exhibited deterioration in the blood flow of the brain, predominantly in the left thalamus (Fig. 3A), and the regional blood flow showed little change, or rather decrease, even after the start of cilostazol treatment (Fig. 3B).

## Discussion

Magnetic resonance imaging and SPECT imaging of the brain of a patient with thalamic pain exhibited clear stroke‐derived lesions and decreased blood flow over an area of the left thalamus, respectively. In addition, stroke‐derived lesions caused severe pain on the right‐side extremities. The left and right reversed relationship between the thalamic lesion and the extremity pain is consistent with an ascending pathway of pain information, indicating that pain in the extremities was derived from thalamic lesions.

This is the first study to show that thalamic pain was markedly alleviated by administration of cilostazol, an antiplatelet, suggesting that some antiplatelets may have analgesic effects. On the other hand, there was little change in the pain score after single administration of amitriptyline or combinational administration of amitriptyline and clomipramine, or amitriptyline and ifenprodil, indicating that these agents have little effect on thalamic pain; therefore, the present study demonstrated that only cilostazol has the analgesic effect. Thus, this finding suggests that pharmacological characteristics of cilostazol elicit an ameliorative effect on thalamic pain; however, the possibility that combinational administration of cilostazol and amitriptyline ameliorated his thalamic pain cannot be ruled out. Similarly, cilostazol administration improved intermittent claudication, including lumbar spinal cord stenosis and peripheral arterial disease 10; however, the underlying mechanisms remain poorly understood.

The normal brain functioning is maintained by the blood–brain barrier consisting of tight junction between adjacent brain endothelial cells, pericytes, astrocytic endfeet, and extracellular matrix components, and it prevents the entry of compounds from blood to brain 11. Hemorrhagic stroke in the thalamus is characterized by structural disruption of tight junction between endothelial cells and increased permeability. Therefore, with a stroke, chemicals and fluid extravasate into brain across the impaired blood–brain barrier as a result of increased permeability and gross lesioning of the endothelium 12. Thus, stroke may enable therapeutic agents such as cilostazol to reach only the lesioning brain region such as the thalamus hemorrhagic stroke, and cilostazol reduces hemorrhagic stroke by protecting the entire blood–brain barrier 13.

Cilostazol is a selective and potent inhibitor of phosphodiesterase (PDE) 3A, which limits the intracellular cAMP levels by catalyzing hydrolysis of cAMP to inactive 5′‐AMP. Thus, it is likely that cilostazol increases the level of cAMP 14; however, the downstream intracellular mechanisms regulated by the increase in cAMP remain to be fully elucidated. A well‐known downstream effect of cAMP is caused by activation of protein kinase A, which leads to intracellular activation by phosphorylating cAMP‐response element‐binding protein. Alternatively, the exchange protein directly activated by cAMP (Epac), another downstream signaling molecule of cAMP, is activated by binding cAMP and is likely a crucial factor in cAMP signaling in many areas of the body 15. Indeed, Epac‐specific signaling properties contribute to the molecular signaling network of cAMP, which regulates glucose homeostasis, cardiac output, vascular functioning, and neuronal processes 16. The increase in cAMP following the administration of cilostazol has antiplatelet and vasodilating effects through its action on smooth muscle cells and in turn works to improve intermittent claudication through the inhibition of platelet aggregation or vasodilation 9. On the other hand, cilostazol caused little change in the thalamic blood flow, indicating that the improvement in the pain score by cilostazol is derived from functions other than the vascular functioning in Epac. Thalamic pain is caused by a loss of inhibition or by increased facilitation in thalamic neurons 5, indicating the importance of thalamic neurons in thalamic pain. The increase in cAMP following the administration of cilostazol results in the activation of Epac, which reduces neuronal cell death, indicating that Epac rescues neurons from cell death 17. Accumulating evidence demonstrates that stroke triggers axonal sprouting in the cortex and the spinal cord, and the axonal sprouting is associated with the recovery of neuronal function 18. Similarly, it is natural that the axonal sprouting is induced following hemorrhage stroke in the thalamus. Judging from the effect of cilostazol on a patient with thalamic pain, it is possible that administration of cilostazol enhances axonal sprouting and, consequently, the function of inhibitory neurons in the thalamus is recovered and thalamic pain is alleviated, although the relationship between axonal sprouting and cAMP remains uncertain. On the other hand, the activation of Epac by the increase in cAMP removes synaptic GluR2/3‐containing AMPA receptors, indicating that excitatory glutamatergic transmission is suppressed 19 and that the pain score is ameliorated as a consequence. Thus, the present data showing that cilostazol, an inhibitor of PDE3, elicited a functional effect on thalamic pain suggests that other PDE3 inhibitors (i.e., anagrelide and milrinone) may also be effective for treating thalamic pain.

Little is known about the underlying mechanisms exerting the effect of cilostazol on thalamic pain. Thus, more clinical trials are needed to establish an evidence‐based treatment of thalamic pain by cilostazol.

## Authorship

AH‐T and HF: conceived and designed the study. AH‐T, HF, YM, and TM: acquired the data. AH‐T, TN, and YI: participated in drafting the manuscript.

## Conflict of Interest

The authors declare no conflicts of interest in association with the present study.
